# Redetermination of phenyl­hydrazinium chloride

**DOI:** 10.1107/S1600536807066974

**Published:** 2007-12-21

**Authors:** Geraldo M. de Lima, James L. Wardell, William T. A. Harrison

**Affiliations:** a Departamento de Quimica, UFMG, Campus - Pampulha, 31270-901 Belo Horizonte, Minas Gerais, Brazil; b Department of Chemistry, University of Aberdeen, Meston Walk, Aberdeen AB24 3UE, Scotland

## Abstract

In the redetermined structure [Koo (1965 ▶). *Bull. Chem. Soc. Jpn*, **38**, 286] of the title compound, C_6_H_9_N_2_^+^·Cl^−^, the H atoms have been located and the hydrogen-bonding scheme established. A series of N—H⋯Cl and N—H⋯N hydrogen bonds leads to a layered network parallel to the (010) plane.

## Related literature

For the earlier structure determination, see: Koo (1965 ▶). For a related structure, see: Hammerl *et al.* (2001 ▶). For reference structural data, see: Allen *et al.* (1987 ▶).
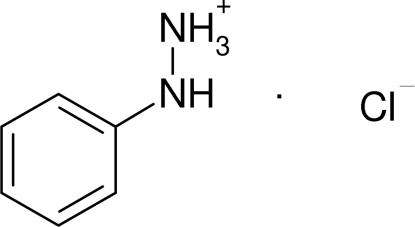


## Experimental

### 

#### Crystal data

C_6_H_9_N_2_^+^·Cl^−^
*M* *_r_* = 144.60
Monoclinic, 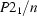

*a* = 3.8223 (5) Å
*b* = 30.461 (5) Å
*c* = 6.0121 (10) Å
β = 100.686 (6)°
*V* = 687.86 (18) Å^3^
*Z* = 4
Mo *K*α radiation
μ = 0.46 mm^−1^
*T* = 120 (2) K
0.30 × 0.07 × 0.01 mm

#### Data collection

Nonius KappaCCD diffractometer
Absorption correction: multi-scan (*SADABS*; Bruker, 2003 ▶) *T*_min_ = 0.874, *T*_max_ = 0.995
3891 measured reflections
1288 independent reflections
1073 reflections with *I* > 2σ(*I*)
*R*_int_ = 0.053

#### Refinement

*R*[*F* ^2^ > 2σ(*F* ^2^)] = 0.072
*wR*(*F* ^2^) = 0.212
*S* = 1.12
1288 reflections
94 parameters
H atoms treated by a mixture of independent and constrained refinement
Δρ_max_ = 0.57 e Å^−3^
Δρ_min_ = −0.62 e Å^−3^

### 

Data collection: *COLLECT* (Nonius, 1998 ▶); cell refinement: *SCALEPACK* (Otwinowski & Minor, 1997 ▶); data reduction: *DENZO* (Otwinowski & Minor, 1997 ▶), *SCALEPACK* and *SORTAV* (Blessing, 1995 ▶); program(s) used to solve structure: *SHELXS97* (Sheldrick, 1997 ▶); program(s) used to refine structure: *SHELXL97* (Sheldrick, 1997 ▶); molecular graphics: *ORTEP-3* (Farrugia, 1997 ▶); software used to prepare material for publication: *SHELXL97*.

## Supplementary Material

Crystal structure: contains datablocks I, global. DOI: 10.1107/S1600536807066974/bt2663sup1.cif

Structure factors: contains datablocks I. DOI: 10.1107/S1600536807066974/bt2663Isup2.hkl

Additional supplementary materials:  crystallographic information; 3D view; checkCIF report

## Figures and Tables

**Table 1:** Hydrogen-bond geometry (Å, °)

*D*—H⋯*A*	*D*—H	H⋯*A*	*D*⋯*A*	*D*—H⋯*A*
N2—H4⋯N1^i^	0.93 (6)	2.11 (6)	3.031 (6)	173 (5)
N1—H1⋯Cl1^ii^	0.90 (6)	2.49 (6)	3.256 (4)	142 (5)
N2—H2⋯Cl1^iii^	1.04 (6)	2.04 (6)	3.079 (4)	176 (5)
N2—H3⋯Cl1	0.90 (7)	2.35 (7)	3.187 (5)	154 (6)

Symmetry codes: (i) 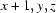; (ii) 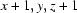; (iii) 
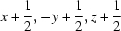
.

**Fig. 1.**
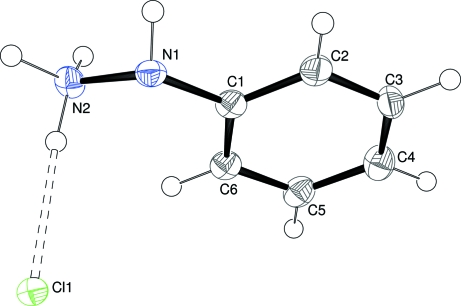
View of the molecular structure of (I) showing 50% displacement ellipsoids. The H atoms are drawn as spheres of arbitrary radius and the hydrogen bond is shown as a double-dashed line.

## supplementary crystallographic information

### Comment

The structure of the title compound, (I), was established by Koo (1965), but no hydrogen atoms were located. The hydrogen bonding pattern in the crystal of (I) cannot be established based on geometrical placement of the H atoms because roatation of the terminal –NH_3_^+^ group leads to different plausible arrangements for the hydrogen bonds. Here, the redetermined structure of (I), including the H atom positions is presented (Fig. 1), and the hydrogen bonding scheme is definitively established.

In (I), atoms N1 and N2 deviate from the C1—C6 ring plane by -0.098 (4)Å and 0.418 (4) Å, respectively. The bond-angle sum for N1 is 331°, indicative of *sp*^3^ hybridization for this atom. Otherwise, the geometrical paramaters for (I) may be regarded as normal (Allen *et al.*, 1987).

The crystal packing for (I) is influenced by cation-to-cation N—H···N and cation-to-anion N—H···Cl hydrogen bonds (Table 1). The former of these leads to [100] chains in the crystal. One of the H···Cl separations is unusually short, with H···Cl = 2.04 (6) Å, which possibly correlates with the its long N—H separation of 1.04 (6) Å. Together, the hydrogen bonds lead to sheets parallel to the (010) plane (Fig. 2).

Only one other crystal structure containing the phenylhydrazinium cation has been determined (Hammerl *et al.*, 2001), which has similar geometrical paramaters to those in (I).

### Experimental

The title compound was prepared by dropwise addition of concentrated hydrochloric acid (1 equivalent) to an ethanolic solution of phenylhydrazine. The product, which appreared on standing, was collected and colourless blades of (I) were recrystallized from EtOH, m.p 525–528 K (decomp).

### Refinement

The N-bound H atoms were located in a difference map and their positions were freely refined with the constraint *U*_iso_(H) = 1.2*U*_eq_(N). The C-bound H atoms were placed in calculated positions (C—H = 0.93 Å) and refined as riding with *U*_iso_(H) = 1.2*U*_eq_(C).

### Crystal data

**Table:**

C_6_H_9_N_2_^+^·Cl^−^	*F*(000) = 304
*M**_r_* = 144.60	*D*_x_ = 1.396 Mg m^−^^3^
Monoclinic, *P*2_1_/*n*	Mo *K*α radiation, λ = 0.71073 Å
Hall symbol: -P 2yn	Cell parameters from 1173 reflections
*a* = 3.8223 (5) Å	θ = 2.9–27.5°
*b* = 30.461 (5) Å	µ = 0.46 mm^−^^1^
*c* = 6.0121 (10) Å	*T* = 120 K
β = 100.686 (6)°	Blade, colourless
*V* = 687.86 (18) Å^3^	0.30 × 0.07 × 0.01 mm
*Z* = 4	

### Data collection

**Table:**

Nonius KappaCCD diffractometer	1288 independent reflections
Radiation source: fine-focus sealed tube	1073 reflections with *I* > 2σ(*I*)
graphite	*R*_int_ = 0.053
ω and φ scans	θ_max_ = 26.0°, θ_min_ = 3.5°
Absorption correction: multi-scan (*SADABS*; Bruker, 2003)	*h* = −4→4
*T*_min_ = 0.874, *T*_max_ = 0.995	*k* = −35→37
3891 measured reflections	*l* = −6→7

### Refinement

**Table:**

Refinement on *F*^2^	Primary atom site location: structure-invariant direct methods
Least-squares matrix: full	Secondary atom site location: difference Fourier map
*R*[*F*^2^ > 2σ(*F*^2^)] = 0.072	Hydrogen site location: inferred from neighbouring sites
*wR*(*F*^2^) = 0.212	H atoms treated by a mixture of independent and constrained refinement
*S* = 1.12	*w* = 1/[σ^2^(*F*_o_^2^) + (0.0789*P*)^2^ + 3.5247*P*] where *P* = (*F*_o_^2^ + 2*F*_c_^2^)/3
1288 reflections	(Δ/σ)_max_ = 0.001
94 parameters	Δρ_max_ = 0.57 e Å^−^^3^
0 restraints	Δρ_min_ = −0.62 e Å^−^^3^

### Special details

**Table:**

Geometry. All e.s.d.'s (except the e.s.d. in the dihedral angle between two l.s. planes) are estimated using the full covariance matrix. The cell e.s.d.'s are taken into account individually in the estimation of e.s.d.'s in distances, angles and torsion angles; correlations between e.s.d.'s in cell parameters are only used when they are defined by crystal symmetry. An approximate (isotropic) treatment of cell e.s.d.'s is used for estimating e.s.d.'s involving l.s. planes.
Refinement. Refinement of *F*^2^ against ALL reflections. The weighted *R*-factor *wR* and goodness of fit *S* are based on *F*^2^, conventional *R*-factors *R* are based on *F*, with *F* set to zero for negative *F*^2^. The threshold expression of *F*^2^ > σ(*F*^2^) is used only for calculating *R*-factors(gt) *etc*. and is not relevant to the choice of reflections for refinement. *R*-factors based on *F*^2^ are statistically about twice as large as those based on *F*, and *R*- factors based on ALL data will be even larger.

### Fractional atomic coordinates and isotropic or equivalent isotropic displacement parameters (Å^2^)

**Table:**

	*x*	*y*	*z*	*U*_iso_*/*U*_eq_	
C1	0.2381 (11)	0.12905 (16)	0.7787 (9)	0.0198 (11)	
C2	0.1470 (12)	0.10308 (16)	0.9478 (9)	0.0230 (11)	
H2A	0.0795	0.1161	1.0771	0.028*	
C3	0.1557 (13)	0.05785 (17)	0.9259 (9)	0.0244 (11)	
H3A	0.0939	0.0398	1.0416	0.029*	
C4	0.2530 (13)	0.03857 (17)	0.7383 (9)	0.0268 (12)	
H4A	0.2574	0.0075	0.7245	0.032*	
C5	0.3432 (13)	0.06488 (17)	0.5721 (9)	0.0253 (12)	
H5	0.4120	0.0519	0.4432	0.030*	
C6	0.3349 (12)	0.11030 (16)	0.5904 (9)	0.0212 (11)	
H6	0.3955	0.1283	0.4741	0.025*	
N1	0.1991 (10)	0.17567 (13)	0.8023 (7)	0.0195 (9)	
H1	0.262 (15)	0.1852 (18)	0.947 (11)	0.023*	
N2	0.4198 (11)	0.20052 (14)	0.6761 (8)	0.0204 (10)	
H2	0.384 (15)	0.234 (2)	0.707 (10)	0.031*	
H3	0.303 (17)	0.1992 (18)	0.532 (12)	0.031*	
H4	0.652 (17)	0.190 (2)	0.712 (10)	0.031*	
Cl1	−0.2104 (3)	0.20115 (4)	0.2468 (2)	0.0192 (4)	

### Atomic displacement parameters (Å^2^)

**Table:**

	*U*^11^	*U*^22^	*U*^33^	*U*^12^	*U*^13^	*U*^23^
C1	0.013 (2)	0.021 (2)	0.023 (3)	0.0028 (17)	−0.0015 (19)	0.0009 (19)
C2	0.017 (2)	0.024 (3)	0.026 (3)	−0.0009 (18)	−0.002 (2)	0.002 (2)
C3	0.024 (3)	0.025 (3)	0.024 (3)	−0.0042 (19)	0.003 (2)	0.007 (2)
C4	0.026 (3)	0.021 (3)	0.034 (3)	0.003 (2)	0.006 (2)	0.001 (2)
C5	0.021 (3)	0.025 (3)	0.030 (3)	−0.001 (2)	0.003 (2)	−0.002 (2)
C6	0.014 (2)	0.024 (2)	0.025 (3)	0.0012 (18)	0.0011 (19)	0.000 (2)
N1	0.018 (2)	0.022 (2)	0.017 (2)	−0.0013 (15)	0.0005 (17)	−0.0011 (17)
N2	0.013 (2)	0.022 (2)	0.025 (3)	0.0040 (16)	−0.0003 (17)	0.0046 (18)
Cl1	0.0187 (6)	0.0199 (6)	0.0182 (7)	−0.0015 (4)	0.0015 (4)	−0.0010 (4)

### Geometric parameters (Å, °)

**Table:**

C1—C6	1.378 (7)	C5—C6	1.389 (7)
C1—C2	1.383 (7)	C5—H5	0.9500
C1—N1	1.438 (6)	C6—H6	0.9500
C2—C3	1.385 (7)	N1—N2	1.449 (6)
C2—H2A	0.9500	N1—H1	0.90 (6)
C3—C4	1.382 (8)	N2—H2	1.04 (6)
C3—H3A	0.9500	N2—H3	0.90 (7)
C4—C5	1.374 (8)	N2—H4	0.93 (6)
C4—H4A	0.9500		
			
C6—C1—C2	120.6 (5)	C6—C5—H5	119.6
C6—C1—N1	122.6 (4)	C1—C6—C5	119.4 (5)
C2—C1—N1	116.6 (4)	C1—C6—H6	120.3
C1—C2—C3	119.0 (5)	C5—C6—H6	120.3
C1—C2—H2A	120.5	C1—N1—N2	112.5 (4)
C3—C2—H2A	120.5	C1—N1—H1	113 (4)
C4—C3—C2	121.0 (5)	N2—N1—H1	105 (4)
C4—C3—H3A	119.5	N1—N2—H2	108 (3)
C2—C3—H3A	119.5	N1—N2—H3	104 (4)
C5—C4—C3	119.2 (5)	H2—N2—H3	99 (5)
C5—C4—H4A	120.4	N1—N2—H4	109 (4)
C3—C4—H4A	120.4	H2—N2—H4	116 (5)
C4—C5—C6	120.7 (5)	H3—N2—H4	120 (6)
C4—C5—H5	119.6		
			
C6—C1—C2—C3	0.2 (7)	C2—C1—C6—C5	−0.5 (7)
N1—C1—C2—C3	175.6 (4)	N1—C1—C6—C5	−175.5 (4)
C1—C2—C3—C4	−0.1 (7)	C4—C5—C6—C1	0.6 (7)
C2—C3—C4—C5	0.2 (7)	C6—C1—N1—N2	−27.3 (6)
C3—C4—C5—C6	−0.5 (7)	C2—C1—N1—N2	157.5 (4)

### Hydrogen-bond geometry (Å, °)

**Table:**

*D*—H···*A*	*D*—H	H···*A*	*D*···*A*	*D*—H···*A*
N2—H4···N1^i^	0.93 (6)	2.11 (6)	3.031 (6)	173 (5)
N1—H1···Cl1^ii^	0.90 (6)	2.49 (6)	3.256 (4)	142 (5)
N2—H2···Cl1^iii^	1.04 (6)	2.04 (6)	3.079 (4)	176 (5)
N2—H3···Cl1	0.90 (7)	2.35 (7)	3.187 (5)	154 (6)

Symmetry codes: (i) *x*+1, *y*, *z*; (ii) *x*+1, *y*, *z*+1; (iii) *x*+1/2, −*y*+1/2, *z*+1/2.

## References

[bb1] Allen, F. H., Kennard, O., Watson, D. G., Brammer, L., Orpen, A. G. & Taylor, R. (1987). *J. Chem. Soc. Perkin Trans. 1*, pp. S1–19.

[bb2] Blessing, R. H. (1995). *Acta Cryst.* A**51**, 33–38. 10.1107/s0108767394005726 7702794

[bb3] Bruker (2003). *SADABS* Bruker AXS Inc., Madison, Wisconsin, USA.

[bb4] Farrugia, L. J. (1997). *J. Appl. Cryst.* **30**, 565.

[bb5] Hammerl, A., Holl, G., Kaiser, M., Klapötke, T. M., Mayer, P., Nöth, H. & Warchhold, M. (2001). *Z. Anorg. Allg. Chem.* **627**, 1477–1482.

[bb6] Koo, C. H. (1965). *Bull. Chem. Soc. Jpn*, **38**, 286.

[bb7] Nonius (1998). *COLLECT* Nonius BV, Delft, The Netherlands.

[bb8] Otwinowski, Z. & Minor, W. (1997). *Methods in Enzymology*, Vol. 276, *Macromolecular Crystallography*, Part A, edited by C. W. Carter Jr & R. M. Sweet, pp. 307–326. New York: Academic Press.

[bb9] Sheldrick, G. M. (1997). *SHELXS97* and *SHELXL97* University of Göttingen, Germany.

